# Foundations of a temporal RL

**Published:** 2023-02-20

**Authors:** Marc W. Howard, Zahra G. Esfahani, Bao Le, Per B. Sederberg

**Affiliations:** Boston University; Boston University; University of Virginia; University of Virginia

## Abstract

Recent advances in neuroscience and psychology show that the brain has access to timelines of both the past and the future. Spiking across populations of neurons in many regions of the mammalian brain maintains a robust temporal memory, a neural timeline of the recent past. Behavioral results demonstrate that people can estimate an extended temporal model of the future, suggesting that the neural timeline of the past could extend through the present into the future. This paper presents a mathematical framework for learning and expressing relationships between events in continuous time. We assume that the brain has access to a temporal memory in the form of the real Laplace transform of the recent past. Hebbian associations with a diversity of synaptic time scales are formed between the past and the present that record the temporal relationships between events. Knowing the temporal relationships between the past and the present allows one to predict relationships between the present and the future, thus constructing an extended temporal prediction for the future. Both memory for the past and the predicted future are represented as the real Laplace transform, expressed as the firing rate over populations of neurons indexed by different rate constants *s*. The diversity of synaptic timescales allows for a temporal record over the much larger time scale of trial history. In this framework, temporal credit assignment can be assessed *via* a Laplace temporal difference. The Laplace temporal difference compares the future that actually follows a stimulus to the future predicted just before the stimulus was observed. This computational framework makes a number of specific neurophysiological predictions and, taken together, could provide the basis for a future iteration of RL that incorporates temporal memory as a fundamental building block.

Consider the experience of listening to a familiar melody. As the song unfolds, notes feel as if they recede away from the present, an almost spatial experience. According to Husserl (1966) “points of temporal duration recede, as points of a stationary object in space recede when I ‘go away from the object.”’ For a familiar melody, Husserl (1966) argues that events predicted in the future also have an analogous spatial extent, a phenomenon he referred to as *protention*. This experience is consistent with the hypothesis that the brain maintains an inner timeline extending from the distant past towards the present and from the present forwards into the future. In addition to introspection and phenomenological analysis, one can reach similar conclusions from examination of data in carefully controlled cognitive psychology experiments (Tiganj, Singh, Esfahani, & Howard, 2022).

The evolutionary utility of an extended timeline for future events is obvious. Knowing what will happen when in the future allows for selection of an appropriate action in the present. Indeed, much of computational neuroscience presumes that the fundamental goal of the cortex is to predict the future (Clark, 2013; Friston & Kiebel, 2009; Friston, 2010; Rao & Ballard, 1999; Palmer, Marre, Berry, & Bialek, 2015).

In AI, a great deal of research focuses on reinforcement learning (RL) algorithms that attempt to optimize future outcomes within a particular planning horizon (Ke et al., 2018; Dabney et al., 2020) without a temporal memory. From the perspective of psychology, RL is a natural extension of the Rescorla-Wagner model (Rescorla & Wagner, 1972) an associative model for classical conditioning (Sutton & Barto, 1981; Schultz, Dayan, & Montague, 1997; Waelti, Dickinson, & Schultz, 2001). Associative models describe connections between a pair of stimuli (or stimulus and an outcome etc) as a simple scalar value. Variables that affect the strength of an association, such as the number of pairings between stimuli, or attention, etc, all combine to affect a single scalar value. Thus, although the strength of an association can fall off with the time between stimuli, the association itself does not actually convey information about time *per se* (Gallistel, 2021a).

In RL, the goal of the Bellman equation is to estimate discounted future reward:

(1) $$V\left(t\right)≃\sum_{\tau }\gamma^{\tau }r\left(t+\tau \right)$$

but without ever explicitly estimating the future r(t+τ). Temporal difference (TD) learning requires only measurement of the reward in the present and the value of states local in time:

(2) $$\delta (t)=r(t)+\gamma \overset{ˆ}{V}(t+1)-\overset{ˆ}{V}(t)$$

In recent years, several authors have pursued temporal alternatives to TD learning (Ludvig, Sutton, & Kehoe, 2008; Kurth-Nelson & Redish, 2009; Momennejad & Howard, 2018; Tiganj, Gershman, Sederberg, & Howard, 2019; Tano, Dayan, & Pouget, 2020). Those models have attempted to incorporate temporal information into states (Ludvig et al., 2008) or choose a spectrum of discount rates to give appropriate behavior at a range of scales (Kurth-Nelson & Redish, 2009; Momennejad & Howard, 2018; Tano et al., 2020). To the extent that the brain can directly estimate the future, the problem solved by the Bellman equation—compute expected future reward without explicitly computing the future—is not a problem that is faced by the brain.

Within psychology, many theorists argue that classical conditioning reflects explicit storage and retrieval of temporal contingencies between stimuli (Cohen & Eichenbaum, 1993; Arcediano, Escobar, & Miller, 2005; Balsam & Gallistel, 2009; Gallistel, Craig, & Shahan, 2019). Recent neurophysiological work (Jeong et al., 2022) has shown that temporal contingency provides a better account of the firing of dopamine neurons than TDRL, highlighting the need for a neural theory for learning temporal contingency. Such a theory requires a temporal memory.

## Temporal memory in the brain

There is overwhelming evidence that temporal memory is widespread throughout the mammalian brain. From examining ongoing neural activity, it is possible to decode what happened when in the past from many brain regions (King & Dehaene, 2014; Murray et al., 2017; Terada, Sakurai, Nakahara, & Fujisawa, 2017; Rossi-Pool et al., 2019; Enel, Wallis, & Rich, 2020; Cueva et al., 2020). There are at least two forms of coding for time that support this ability. So-called time cells (Pastalkova, Itskov, Amarasingham, & Buzsaki, 2008; MacDonald, Lepage, Eden, & Eichenbaum, 2011, for reviews see Eichenbaum, 2014, 2017; Tsao, Yousefzadeh, Meck, Moser, & Moser, 2022) fire in sequence following a salient stimulus. Different stimuli trigger different sequences of time cells, so that from observing which time cells are firing at any moment it is is possible to decode what happened when in the past. In addition, so-called “temporal context cells” are triggered shortly after presentation of an event and then relax exponentially back to their baseline firing rate. Critically, temporal context cells have a heterogeneity of time constants (Tsao et al., 2018; Bright et al., 2020). Because different stimuli trigger different temporal context cells and because temporal context cells have a wide range of time constants, one can decode information about what happened when in the past over a wide range of time scales from a population of temporal context cells.

**Figure 1.**
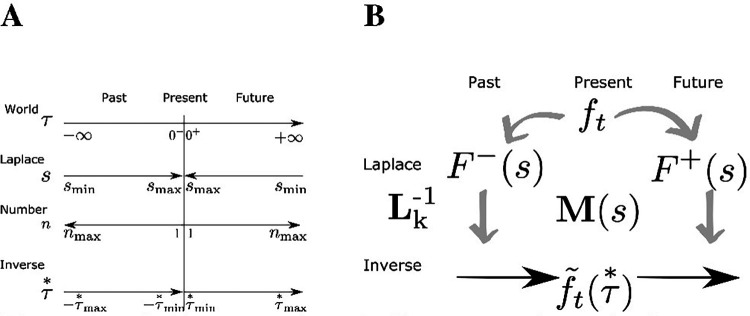
Guide to notation. **A.** Sign conventions. At the present moment t, objective time τ runs from −∞ to ∞. τ=0 corresponds to time t. The real Laplace domain variable s runs from $0^{+}$ to +∞ for both past and future, approximated as $s_{min}$ and $s_{max}$. The units of s are $t^{-1}$; the values corresponding to different points of the timeline are shown in the same vertical alignment. Cell number for Laplace and inverse spaces n are aligned with one another. The variable *taustar* describes position along the inverse spaces. It is in register with τ and derived from s. **B.** The stimulus available in the present, $f_{t}$ provides input to two sets of neural manifolds. One set of neural manifolds represents the past; the other estimates the future. M(s) stores temporal relationships between events.

The properties of temporal context cells—exponential receptive fields with a wide range of time constants—are as one would expect if firing rate across the population of temporal context cells records the real Laplace transform of the past leading up to the present (Shankar & Howard, 2010; Howard et al., 2014). Let’s assume that f(t) is a vector describing which of several discrete events happen at time t and that f(t) is zero for most values of t. We can specify the past leading up to each moment t as $f_{t}(\tau )$ (see Figure 1) with τ ranging from zero to −∞ describing how far in the past an event was experienced. The goal of the temporal memory is to estimate the past $f_{t}(\tau )$ at each time t,

(3) $$F_{t}(s)=\int_{0}^{\infty }\;e^{-s\tau }f_{t}(-\tau )d\tau =ℒ\left\{f_{t}(-\tau )\right\}(s)$$

where we understand s to be restricted to the positive real line. The observation that $F_{t}(s)$ is the Laplace transform of $f_{t}(-\tau )$ establishes that it serves as a temporal memory. Time cells, with circumscribed receptive fields resemble a “direct” estimate of the past:

(4) $${\tilde{f}}_{t}(\overset{*}{\tau }<0)=-\frac{1}{\overset{*}{\tau }}\int_{0}^{\infty }\;\Phi \left(\frac{-\tau }{{\;}_{\tau }^{*}}\right)f_{t}(-\tau )d\tau$$

where Φ(x) is a unimodal function with its maximum at 1 and $\overset{*}{\tau }$ is defined to be negative. Time cells have properties that we would expect if the firing rate over a population of neurons records the approximate inverse Laplace transform. As Φ() becomes more and more sharp, approaching a delta function, we see that ${\tilde{f}}_{t}(\overset{*}{\tau })$ goes to $f_{t}(t+\overset{*}{\tau })$. The properties of time cells in the hippocampus confirm several predictions that follow from this hypothesis (Kraus, Robinson, White, Eichenbaum, & Hasselmo, 2013; Taxidis et al., 2020; Cao, Bladon, Charczynski, Hasselmo, & Howard, 2022).

## Eligibility traces and memory in RL

Although RL models have historically assumed Markov statistics and used the theory of Markov decision processes, the idea of a temporal memory is not at all foreign to RL. The eligibility trace (Sutton, 1988) at time t, $e_{t}$ updates from time step to time step as

$$e_{t}=\lambda e_{t-1}+f_{t}$$

where $f_{t}$ is the state observed at time t. It is clear that this expression results in exponential forgetting of inputs. Taking the continuum limit we find

(5) $$e_{t}=\sum_{0}^{\infty }\lambda^{\tau }f_{t-\tau }≃\int_{0}^{\infty }e^{-s\tau }f_{t}\left(\tau \right)d\tau$$

where in the last expression s is chosen as s=−log⁡λ. Thus the eligibility trace is an exponentially-weighted sum over past events. Comparing this last expression to Eq. 3, we find that the salient difference between the eligibility trace and Laplace transform of the past is that the eligibility trace is usually understood to have one forgetting rate λ whereas the Laplace transform requires a continuum of rate constants s.

By choosing a continuum of forgetting rates, one obtains a temporal memory extending roughly from the fastest time constant to the slowest time constant. The resolution of this temporal memory is controlled by the spacing between adjacent time constants and the degree to which the firing rates of spiking neurons can faithfully obey Eq. 3. Critically, the resolution of this Laplace temporal memory does not depend on the properties of Φ() or whatever mechanism is used to extract information. This paper assumes the existence of a population of neurons whose firing rate $F_{t}^{-}(s)$ encodes the real Laplace transform of the past leading up to time t. One of the primary goals of the paper is to develop a hypothesis that allows construction of a population $F_{t}^{+}(s)$ that provides an estimate of the real Laplace transform of the future that is expected to follow time t.

## Constructing neural timelines of the past and future

The goal of this paper is to write out a formalism we can take seriously to describe laboratory behavioral tasks used in psychology and neuroscience. We assume that the input to the model is a finite set of discrete symbols, x, y, etc., that are occasionally presented for an instant in continuous time. We refer to the symbol available at any particular moment as a stimulus. When a symbol is presented at time t, the stimulus is the basis vector for that stimulus for a delta function centered at t. At most times, the stimulus is zero. We assume that there are temporal relationships between some of the symbols. For convenience we assume that the time between repetitions of any symbol is much longer than the temporal relationships that are to be discovered and much longer than the longest time constant $1/s_{min}$. This assumption allows us to imagine that experience is segmented into a series of discrete trials; each symbol can be presented at most once per trial. This assumption is not fundamental to the model but allows easy interpretation of quantities that we will derive.

### The present

Let us take as input to the model a stream of inputs, f(t). The notation v refers to a vector with each element a real number, $v^{\prime }$ is a transposed vector, so that $u^{\prime }v$ is the inner product, a scalar, and ${uv}^{\prime }$ is the outer product, a matrix. We write $f_{t}$ for the stimulus available at time t. At instants t when no stimulus is presented, $f_{t}=0$, the vector with all entries zero. We will occasionally refer to the moment on a particular trial when x is presented, $f_{t}=x$ as $t_{x}$. We ignore similarity between symbols, so that $y^{\prime }x=\delta_{y,x}$. If symbol x was predicted to occur at time t, but was not observed, we will occasionally write $f_{t}=\tilde{x}$ to describe the observation of a failed prediction for symbol x. One may imagine the basis vectors x, y, $\tilde{x}$ etc as one-hot vectors without changing any of the results in this paper.

We write $f_{t}(\tau )$ to describe the true past that led up to time t, where τ runs from $0^{-}$, corresponding to the moment of the past closest to the present backwards to −∞, corresponding to the distant past. Whereas $f_{t}$ is the stimulus available in the present at time t, $f_{t}(\tau <0)$ is the timeline that led up to time t. Under the assumption that every symbol is presented at most once per trial, each component of $f_{t}(\tau <0)$ is either a delta function at some particular τ or zero everywhere.

### Neural manifolds for the past and the future

We estimate both the past and the future as functions over neural manifolds. Each manifold is a population of processing elements—neurons—each of which is indexed by a position in a coordinate space. The coordinates describing the neurons are continuous and locally Euclidean. At each moment, each neuron is mapped onto scalar value correspond to its firing rate over a macroscopic period of time on the order of at least tens of ms. We propose that the past and the future are represented by separate manifolds that interact with one another.

The representations for both the past and the future each utilize two connected manifolds. We refer to one kind of manifold, indexed by an effectively continuous variable s, as a Laplace space. The other kind of manifold, indexed by an effectively continuous variable $\overset{*}{\tau }$, is referred to as an inverse space. The representations of the past follow previous work in theoretical neuroscience (Shankar & Howard, 2013; Howard et al., 2014), psychology (Howard, Shankar, Aue, & Criss, 2015), and neuroscience (Bright et al., 2020; Cao et al., 2022).

#### Laplace spaces for remembered past and predicted future.

The Laplace space corresponding to the past, which we write as $F_{t}^{-}(s)$ encodes the Laplace transform of $f_{t}(\tau )$, the past leading up to time t:

(6) $$F_{I}^{-}(s)=ℒ\left\{f_{t}(\tau <0)\right\}(s)$$

We restrict s to real values on the positive line (but see Aghajan, Kreiman, & Fried, 2022). The Laplace space corresponding to the future, which we write as F^+^(*s*) is an an attempt to estimate the Laplace transform of the future, $ℒ\left\{f_{t}(\tau >0)\right\}(s)$. Many neurons tile the s axis continuously for each symbol. One may imagine that each symbol, rather than being represented by a single neuron as in a one-hot vector, is represented by a line of neurons representing the history, or future, of that symbol. The index s assigned to a neuron corresponds to the inverse of its functional time constant. Thus, there is a natural mapping between 1/s and τ within both the past and the future. By convention, s is positive for both the past and the future so that $F_{t}^{-}(s)$ is the Laplace transform of $f_{t}(-\tau )$ for τ<0 whereas $F_{t}^{+}(s)$ is the Laplace transform of $f_{t}(\tau )$ for τ>0.

Although s is effectively continuous, this does not require that neurons sample s evenly. Following previous work in psychology (e.g., Chater & Brown, 2008; Piantadosi, 2016; Howard & Shankar, 2018), neuroscience (Guo, Huson, Macosko, & Regehr, 2021; Cao et al., 2022), and theoretical neuroscience (Lindeberg & Fagerström, 1996; Shankar & Howard, 2013), we assume that s is sampled on a logarithmic scale. Let n be the neuron number, starting from the largest value of $s_{max}$ nearest τ=0 and extending out from the present. We obtain a logarithmic scale by choosing ds/dn=−s.

#### Updating Laplace spaces in real time.

Suppose that we have arranged for one particular component of $F_{t}^{-}(s)$ or $F_{t}^{+}(s)$ to hold the Laplace transform of one particular symbol, which we write as $f_{t}(\tau )$. Suppose further that $f_{t}(\tau )$ is zero in the neighborhood of τ=0. Consider how this component, which we write as $F^{-}(s)$ or $F^{+}(s)$, should update as time passes. Let us pick some minimal increment of time δt on the order of, say, 100 ms. At time t+δt, information in $f_{t}(\tau <0)$ recedes further away from the present, so that $F_{t+\delta t}^{-}(s)=ℒ\left\{f_{t}(\tau +\delta t)\right\}$. In contrast, at time t+δt, information in $f_{t}(\tau >0)$ comes closer to the present, so that $F_{t+\delta t}^{+}(s)=ℒ\left\{f_{t}(\tau -\delta t)\right\}$. More generally, suppose that $F_{t}(s)$ is the Laplace transform of a function over some variable x, $F_{t}(s)=ℒ\left\{f_{t}(x)\right\}(s)$. Defining α≡δx/δt, we can update $F_{t}(s)$ as

(7) $$F_{t+\delta t}(s)=ℒ\left\{{𝒯}_{\alpha (\delta t)}f_{t}(x)\right\}(s)=e^{-s\alpha (\delta t)}F_{t}(s)$$

where 𝒯 is the translation operator, ${𝒯}_{a}f(x)=f(x+a)$ and we have used the expression for the Laplace transform of translated functions. Equation 7 describes a recipe for updating both $F_{t}^{\pm }(s)$ with $\alpha_{\pm }$ in the absence of new input. Using the sign convention developed here, we fix $\alpha_{-}=1$ for $F^{-}(s)$ and fix $\alpha_{+}=-1$ for $F^{+}(s)$. It is possible to incorporate changes into the rate of flow of subjective time by letting $\alpha_{\pm }$ change in register, such that $\alpha_{+}(t)=-\alpha_{-}(t)$ for all t. The expression in Eq. 7 holds more generally and can be used to update Laplace transforms over many continuous variables of interest for cognitive neuroscience (Howard et al., 2014; Howard, Luzardo, & Tiganj, 2018; Howard & Hasselmo, 2020).

We are in a position to explain how $F_{t}^{-}(s)$ comes to represent the Laplace transform of $f_{t}(\tau <0)$; a discussion of how $F_{t}^{+}(s)$ comes to estimate the future requires more development and will be postponed. When a symbol is presented at time t, it enters timeline of the past at $\tau =0^{-}$. So, incorporating the input at time t into the past at time t+δt we have

(8) $$F_{t+\delta t}^{-}(s)\;=e^{-s(\delta t)}\left[F_{t}^{-}(s)+ℒ\left\{\delta \left(0^{-}\right)f_{t}\right\}\right]\;\;=e^{-s(\delta t)}F_{t}^{-}(s)+e^{-s(\delta t)}f_{t}.$$

At time t+δt, the input from time t is encoded as the Laplace transform of that symbol a time δt in the past. At each subsequent time step, an additional factor of $e^{-s\delta t}$ accumulates. As time passes, the input from time t is always stored as Laplace transform of a delta function at the appropriate place on the timeline. Because this is true for all stimuli that enter $F^{-}(s)$, we conclude that $F_{t}^{-}(s)$ encodes the Laplace transform of the past $f_{t}(\tau <0)$.

**Figure 2.**
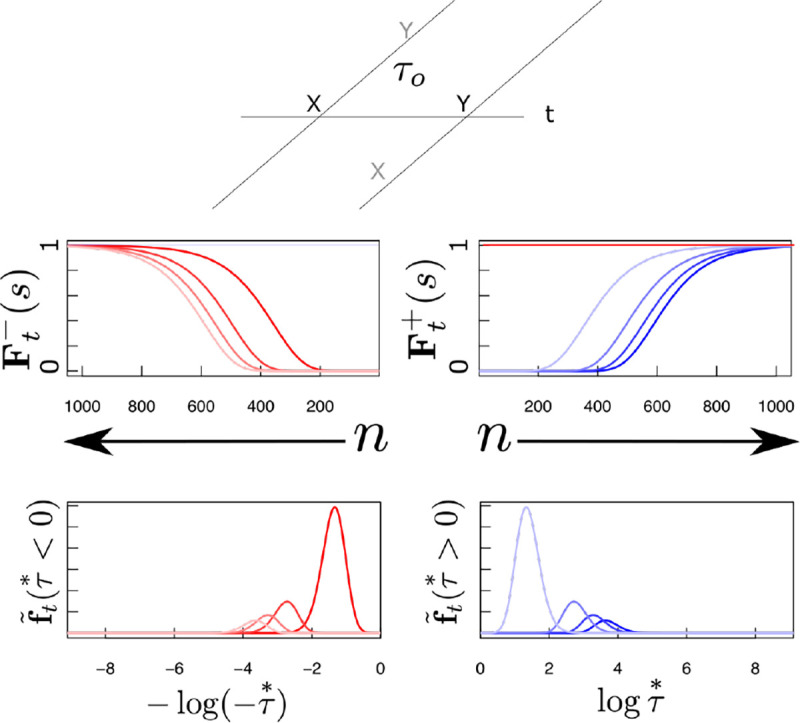
Neural manifolds to construct a log compressed timeline of the past and the future. Top: A temporal relationship exists between x and y such that y always follows y after a delay of $\tau_{o}$ seconds. Consider how the internal timeline ought to behave after x is presented at t=0. At time t, the past should include xt seconds in the past and y $\tau_{o}-t$ seconds in the future. Samples of the timeline at evenly-spaced moments between zero and $\tau_{o}$. Earlier moments closer to t=0 are darker and later moments closer to $t\;=\tau_{o}$ are lighter. Red lines are neurons coding for x, blue lines are neurons coding for y. Middle: Laplace spaces for the past (left) and future (right) shown as a function of cell number n; Bottom: inverse spaces, constructed using the Post approximation, for the past (left) and future (right) shown as a function of log time. Exactly at time t=0, x is available a time $0^{+}$ in the future (dark horizontal red line, middle right). Similarly, exactly at $t=\tau_{o}$, y is available a time $0^{-}$ in the past (light horizontal blue line, middle left).

The middle panel of Figure 2 illustrates the profile of activity over $F_{t}^{-}$ and $F_{t}^{+}$, shown as a function of cell number n, resulting from the Laplace transform of a delta function at various moments in time. In the middle panel, the axis for the past is reversed to allow appreciation of the relationship between past time τ<0 and $F^{-}$. Note that the Laplace transform of a delta function has a characteristic shape as a function of cell number that merely translates as time passes. Note that the magnitude of the translation of $F^{\pm }[n]$ depends on the value of $\tau_{o}$. It can be shown that for a delta function $F_{t+\delta t}^{\pm }[n]=F_{t}^{\pm }[n+\delta n]$ with $\delta n=\alpha_{\pm }\frac{\delta t}{\tau_{o}}$. This can be appreciated by noting that the distances between successive lines in the middle panel of Fig. 2 are not constant despite the fact that they correspond to the same time displacement. Whereas δn goes down as time passes for $F^{-}[n],$ δn increases with the passage of time for $F^{+}[n]$.

There are implementational challenges to building a neural circuit that obeys Eq. 7; these challenges are especially serious when α<0, which requires activation to grow exponentially. These challenges would be mitigated by a neural circuit that obeys an equivalent PDE obtained by differentiating Eq. 7 with respect to cell number n. The use of a PDE allows error to be distributed over many neurons and would allow neurons that have zero activation to grow if their neighbors are nonzero. Moreover, if one could literally implement the PDE this would preserve linearity. A disadvantage of a PDE is that it may require careful tuning of a neural circuit. If one were willing to restrict the representation of each symbol to the Laplace transform of a delta function at a single point in time, it would be straightforward to implement a continuous attractor network (Khona & Fiete, 2021) to allow the “edge” in the Laplace transform as a function of n to translate appropriately.

### Inverse spaces for remembered past and predicted future.

The bottom panel of Figure 2 shows a graphical depiction of the inverse space for the past and the future during the interval between presentation of x and y. The inverse spaces approximate the past, $\tilde{f}(\overset{*}{\tau }<0)$, and the future, $\tilde{f}(\overset{*}{\tau }>0)$, on a log scale. Neurons in the inverse space have circumscribed receptive fields in time, like time cells in the hippocampus (Pastalkova et al., 2008; MacDonald et al., 2011). As the delta function corresponding to the time of x recedes into the past, the corresponding bump of activity in $x^{\prime }{\tilde{f}}_{t}(\overset{*}{\tau }\;>\;0)$ also moves, keeping its shape but moving more and more slowly as x recedes further and further into the past. In the future, the delta function corresponding to the predicted time of y should start a time $\tau_{o}$ in the future and come closer to the present as time passes. As the prediction for y approaches the present, the corresponding bump of activity in $y^{\prime }{\tilde{f}}_{t}(\overset{*}{\tau }>0)$ keeps its shape but the speed of the bump accelerates rather than slowing with the passage of time.

Previous papers have made use of the Post approximation to implement the inverse transform. This is not neurally reasonable (Gosmann, 2018); the Post approximation is difficult to implement even in artificial neural networks (e.g., Tano et al., 2020; Jacques, Tiganj, Howard, & Sederberg, 2021). A more robust approach would be a continuous attractor network (for a review see Khona & Fiete, 2021) that takes input as the derivative of F with respect to n. The width of the bump in $\tilde{f}$ would depend on internal connections between neurons in $\tilde{f}$ and global inhibition would stabilize the activity over $\tilde{f}$. In this case, moving the bump in different directions, corresponding to α>0 and α<0 is analogous to moving the bump of activity in, say, a ring attractor for the head direction system, in different directions.

### Predicting the future from the past

The previous subsection describes how to evolve the Laplace manifold for the past. We could use the same approach to evolve the Laplace manifold for the future during periods when no symbol is experienced if we could initialize the representation of the future appropriately. This will be accomplished *via* learned temporal relationships between the past and the future. For present we only consider simple pairwise relationships between symbols.

The moment a nonzero stimulus $f_{t}$ is experienced, we assume it is available to both $F^{-}$ and $F^{+}$, triggering a number of operations which presumably occur sequentially within a small window of time on the order of perhaps 100 ms. First, the present stimulus updates a prediction for the future *via* a set of connections M organized by s. Then these connections are updated by associating the past to the present. Finally the present stimulus is added to the representation of the past. For ease of exposition we will first focus on describing the connections between the past and the future.

**Figure 3.**
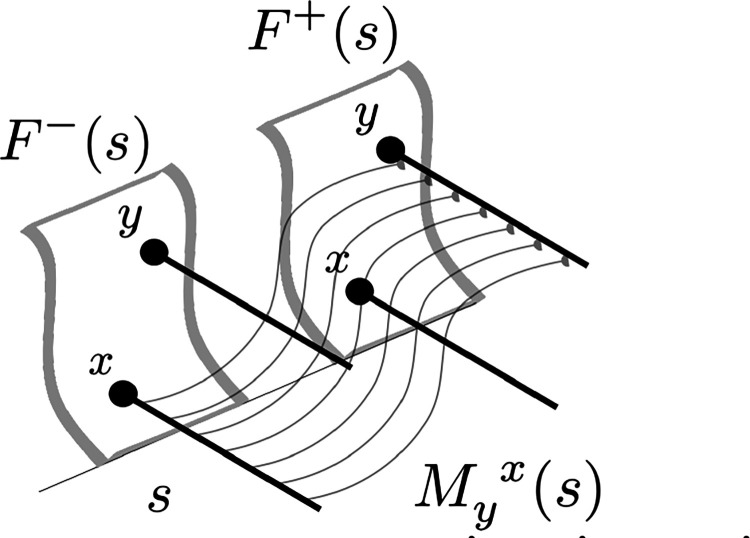
Schematic figure illustrating ${M_{y}}^{x}(s)$. $F^{-}(s)$ and $F^{+}(s)$ components for all the possible symbols, here shown schematically as sheets. Two symbols x and y are shown in both $F^{-}(s)$ and $F^{+}(s)$. Each symbol is associated with a population of neurons spanning a continuous set of s values, shown as the heavy lines in this cartoon. M(s) describes the connections between each symbol in $F^{-}(s)$ to each symbol in $F^{+}(s)$ for each value of s. The curved lines ${M_{y}}^{x}(s)$ illustrate the set of weights connecting units corresponding to x in $F^{-}$ to units corresponding y in $F^{+}$. Connections exist only between units with the same values of s. The strength of the connections in ${M_{y}}^{x}(s)$ vary as a function of s in a way that reflects the pairwise history between x and y.

We write M(s) for a set of connections that associates the Laplace transform of the past to the Laplace transform of the future (Fig. 3). For any particular value $s_{o}$, $M\left(s_{o}\right)$ is a matrix describing connections from each symbol in $F^{-}\left(s_{o}\right)$ to each symbol in $F^{+}\left(s_{o}\right)$. For each pair of symbols, say x and y, we write $M_{y}^{x}\left(s_{o}\right)$ for the strength of the connection *from* the cell corresponding to x with $s=s_{o}$ in $F^{-}$ *to* the cell corresponding to y in $F^{+}$ with $s=s_{o}$. M(s) does not include connections between neurons with different values of s. On occasion it will be useful to think of the set of connections between a pair of symbols over all values of s, which we write as $M_{y}^{x}(s)$. Similarly, we write $M^{y}(s)$ for the set of connections *from* y in $F^{-}$ to all stimuli in $F^{+}$ over all values of s. We write $M_{y}(s)$ for the set of connections *to* y in $F^{+}$ from all symbols and all values of s.

When a particular stimulus, let’s say y, is presented, the connections to and from that stimulus in M(s) are updated. The connections from y in the past are updated as

(9) $$M^{y}(s)\to \rho M^{y}(s)$$

That is, the connections from y in $F^{-}$ to every other stimulus for each value of s, are all scaled down by a value ρ. Later we will consider the implications of a continuous spectrum of ρ values; for now let us just treat ρ as a fixed parameter restricted to be between zero and one. When y is presented, it momentarily becomes available at the “rearward part” of the future. In much the same way that the present enters the past (Eq. 8) at $\tau =0^{-}$, we also assume that the present is also available momentarily in the future at $\tau =0^{+}$. The connections to y in the future are updated as

(10) $$M_{y}(s)\to M_{y}(s)+(1-\rho )F_{t}^{-}(s)$$

**Figure 4.**
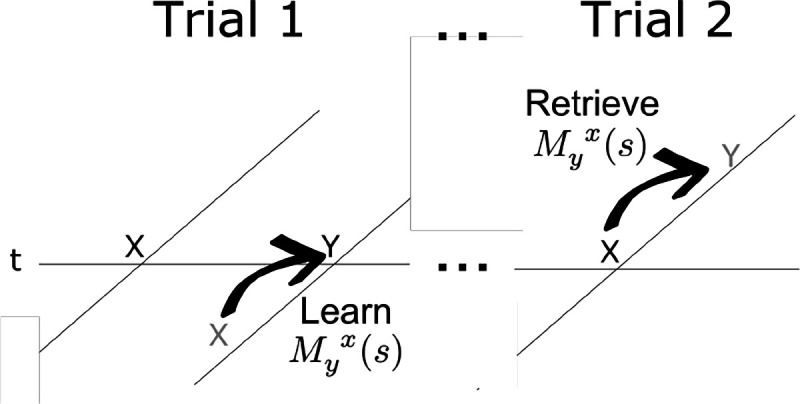
Learning and expressing pairwise associations with M(s). The horizontal line is time; the diagonal lines indicate the internal timeline at the moments they intersect. Memory for the past is below the horizontal line; prediction of the future is above. When x is presented for the first time, it predicts nothing. When y is presented, the past contains a memory for x in the past. When y is presented, ${M_{y}}^{x}(s)$ stores the temporal relationship between x in the past and y in the present—the rearward part of the future. In addition to storing learned relationships, connections from each item decay each time it was presented (not shown). When x is repeated much later in time, the stored connections in ${M_{y}}^{x}(s)$ retrieve a prediction of y in the future.

We can understand Eq. 10 as a Hebbian association between the units in $F^{-}(s)$, whose current activation is given by $F_{t}^{-}(s)$ and the units in the future $F^{+}(s)$ corresponding to the present stimulus y (see Fig. 4). More generally, we can understand this learning rule as strengthening connections from the past $F_{t}^{-}(s)$ to the rearward part of the future, $ℒ\left\{\delta \left(0^{+}\right)f_{t}\right\}(s)=e^{-s0}f_{t}$. Because the second term is the product of two Laplace transforms, it can also be understood as the Laplace transform of a convolution, here, the convolution of the present with the past.^1^ Convolution has long been used as an associative operation in mathematical psychology (Murdock, 1982; Jones & Mewhort, 2007), neural networks (Plate, 1995; Eliasmith, 2013; Blouw, Solodkin, Thagard, & Eliasmith, 2016), and computational neuroscience (Steinberg & Sompolinsky, 2022).

#### M(s) stores pairwise temporal relationships.

To understand the properties of M(s), let us assume that the model as described thus far learns in a world containing two stimuli, x and y for many trials. Let us assume that x is presented on each trial. If y is presented on a given trial it appears precisely $\tau_{o}$ seconds after x. The probability y is presented on each trial is P(y∣x). In this simple situation we can restrict our attention to $M_{y}{\;}^{x}(s)$, the weights connecting the neurons coding for x in the past to the neurons coding for y in the future.

From examination of Eqs. 9 and 10, we see that after each trial ${M_{y}}^{x}(s)$ is multiplied by ρ when x was presented. For trials on which y was also presented, $(1-\rho )e^{-s\tau_{o}}$ is added to ${M_{y}}^{x}(s)$. Writing h[i] as an indicator variable for the history of presentations of y on the trial i steps in the past we find

(11) $${M_{y}}^{x}\left(s\right)=\left(1-\rho \right)e^{-s\tau_{o}}\sum_{i}\rho^{i}h\left[i\right]$$

Note that if P(y∣x)=1, then after an infinitely long series of trials $\sum_{i}\;h[i]\rho^{i}=\frac{1}{1-\rho }$ and ${M_{y}}^{x}(s)=e^{-s\tau_{o}}$ for all choices of ρ. Following similar logic, if we relax the assumption that P(y∣x)=1 and take the limit as ρ goes to 1, we find that ${M_{y}}^{x}(s)=P(y|x)e^{-s\tau_{o}}$.

Now let us relax the assumption that the time lag between x and y always takes the same value. Let the lag be a random variable $\tau_{xy}$ subject to the constraint that $\tau_{xy}$ is always s>0. This is not a fundamental restriction; if $\tau_{xy}$ changed sign, those observations would contribute to ${M_{x}}^{y}(s)$ instead of ${M_{y}}^{x}(s)$. Now, again taking the limit as ρ→1, we find

(12) $${M_{y}}^{x}(s)=P(y|x)E\left[e^{-s\tau_{xy}}\right]=P(y|x)ℒ\left\{\tau_{xy}\right\}(s)$$

where we have used the definition for the Laplace transform of a random variable, again with the understanding that we restrict s to be real and positive.

Equation 12 illustrates several important properties of M(s). First, we can see that ${M_{y}}^{x}(s)$ provides complete information about the distribution of temporal lags averaged over history. This can be further appreciated by noting that the Laplace transform of the random variable on the right hand side is the moment generating function of $-\tau_{xy}=\tau_{yx}$. Keeping the computation in the Laplace domain means that there is no blur introduced by going into the inverse space as in previous attempts to build a model for predicting the future (Momennejad & Howard, 2018; Tiganj et al., 2019; Goh, Ursekar, & Howard, 2022). Second, because $ℒ\left\{\tau_{xy}\right\}\left(s=0\right)=1$ as long as the expectation of $\tau_{xy}$ is finite, we can write ${M_{y}}^{x}(s)={M_{y}}^{x}(s=0){{\overset{ˆ}{M}}_{y}}^{x}(s)\;$ where $M_{y}{\;}^{x}(s=0)$ is just P(y∣x). This allows us to cleanly decompose information about what will happen in the future, stored in ${M_{y}}^{x}(s=0)$, from information about when those events will happen, stored in ${\overset{ˆ}{M}}_{y}{\;}^{x}(s)$ More generally we can express M(s) as $M(s)=M_{\text{what}\;}\cdot \overset{ˆ}{M}(s)$ where · indicates pointwise rather than matrix multiplication.

#### Continuum of ρ *and memory for trial history*.

Before moving on we briefly note the implications of understanding ρ as a continuous variable. Treating ρ as continuous, Eq. 11, which describes the situation where $\tau_{xy}$ is equal to $\tau_{o}$ on each trial, can be rewritten as

$${M_{y}}^{x}\left(\rho ,s\right)=\left(1-\rho \right)e^{-s\tau_{o}}𝒵\left\{h\left[i\right]\right\}\left(\rho^{-1}\right)$$

where 𝒵{}(z) is the Z-transform, the discrete analog of the Laplace transform (Ogata, 1970).

Although the notation is a bit more unwieldy, allowing $\tau_{xy}$ to vary across trials we see that the trial history of timing is also retained by M(ρ,s). Writing the delay between x and y on the trial i steps in the past as τ[i], and $H[i](s)\equiv h[i]e^{-s\tau [i]}$

(13) $$M_{y}^{x}(\rho ,s)=(1-\rho )𝒵\{H[i](s)\}\left(\rho^{-1}\right).$$

**Figure 5.**
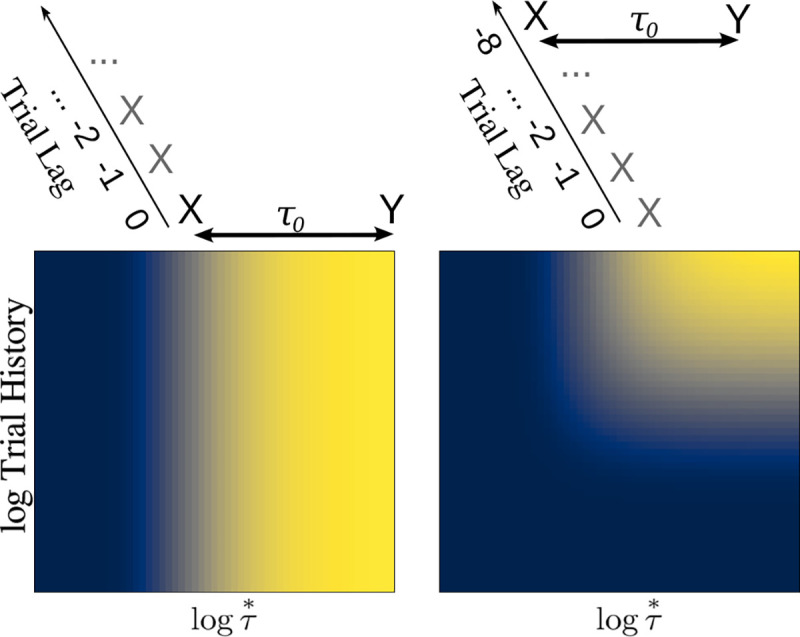
M(ρ,s) contains information about both time within a trial and trial history. Left: Consider a single pairing of x and y on the most recent trial. The heatmap shows the degree to which y is cued by x by $\frac{y^{\prime }M(\rho ,s)x}{1-\rho }$ projected onto log time. The profile as a function of log⁡τ is identical to the profile for future time in Figure 2. If the pairing between x and y had a longer delay, the edge would be further to the right. Right: The single pairing of x and y is followed by an additional series of trials on which x was presented by itself. Now there is an edge in both trial history and time within trial. Additional trials with only x would push this edge further towards the top of the graph. Additional trials with x and y paired would be added to this plot with a time delay that reflects the timing of the pairing.

Because the Z-transform is in principle invertible, information about the entire trial history has been retained by virtue of having a continuum of forgetting rates ρ. Figure 5 illustrates the ability to extract the trial history including timing information of events that follow x from M(ρ,s)x. This illustrates a remarkable property of Laplace-based temporal memory. Although each synaptic matrix with a specific value of $\rho_{o}$ forgets exponentially with rate $-log\rho_{o}$, the set of matrices with a continuum of ρ retains information about the entire trial history. Of course, in practice there must be some numerical imprecision in the biological instantiation of M(ρ,s). In principle however, a continuum of forgetting rates ρ means that the past is not forgotten. Rather the past, here as a function of trial history, has been written to the continuous values of ρ.

### Updating the future

Let us return to the problem of generating a prediction of the immediate future. We again restrict our attention to the limit as ρ goes to 1 and assume the system has experienced a very long sequence of trials with the same underlying statistics. Moreover, we assume for the present that only pairwise relationships are important, so we can neglect the temporal credit assignment problem, and construct the Laplace transform of the future that predicted solely on the basis of the present stimulus.

There are two problems that need to be resolved to write an analog of Eq. 8 for $F_{t+\delta t}^{+}(s)$. First, we can only use use Eq. 7 to update $F_{t}^{+}(s)$ if $F_{t}^{+}(s)$ is already the Laplace transform of a predicted future; we must create a circumstance that makes that true. Second, we need to address the situation where a prediction reaches the present. Because of the discontinuity at τ=0 special considerations are necessary to allow the time of a stimulus to pass from the future to the past.

#### Predicting the future with the present.

Equation 12 indicates that the weights in $M_{y}^{x}(s)$ record the future occurrences of y given that x occurs in the present. $M_{y}^{x}(s)$ captures both the probability that y will follow x as well as the distribution of temporal delays at which y is expected to occur. This information is encoded as a probability times the Laplace transform of a random variable. If we only need to consider x in predicting the future, then $M_{y}^{x}(s)$ is precisely how we would like to initialize the future prediction for y in $F_{t}^{+}(s)$ after x is presented (Fig. 4).

We probe M(s) with the “immediate past.” When x is presented it enters $F_{t}^{-}(s)$ as $ℒ\left\{\delta \left(0^{-}\right)x\right\}(s)$. Multiplying M(s) from the right with the immediate past, yields a prediction for the future.

(14) $$M(s)e^{-s0}x=M(s)x=P(y|x)ℒ\left\{\tau_{xy}\right\}y$$

More generally, the input to the future at time t should be given by $M(s)ℒ\left\{\delta \left(0^{-}\right)f_{t}\right\}$. For concision we write this as $M(s)f_{t}$. Because the past stored in M(s) was a probability times the Laplace transform of a probability distribution, so too will the future recovered in this way.

#### Continuity of the predicted future through τ=0.

The neural representation described here approximates a continuous timeline by stitching together separate Laplace neural manifolds for the past and the future. With the passage of time, information in the future moves ever closer to the present. As time passes and a prediction reaches the present, this discontinuity must be addressed.

We can detect predictions that have reached the present by examining $F_{t}^{+}(s=\infty )$, which only rises from zero when τ→0. In practice, we would use $s_{max}$ which should be on the order of $(\delta t{)}^{-1}$. If the future that is being represented is the Laplace transform of a delta function, then we can simply take components for which $F_{t}^{+}\left(s_{max}\right)>0$ to zero for all s at the next time step. More generally, if the future that is represented is not simply a delta function, the linearity of the Laplace transform allows us to subtract $F_{t}^{+}(s=\infty )$ from all s values without affecting the evolution at subsequent time points.

If a prediction reaches the present and is observed, then no further action is needed. If a prediction reaches the present, but is not observed, we can trigger an observation of a “not stimulus”, written e.g., $\tilde{x}\;$ to describe the observation of a failed prediction for a stimulus x. Although we won’t pursue it here, one could allow “not stimuli” to be predicted by stimuli and to predict other stimuli, allowing for the model to provide appropriate predictions for a relatively complex set of contingencies.

#### Evolving $F_{t+\delta t}^{+}(s)$.

Integrating these two additional factors allows us to write a general expression for evolving $F_{t}^{+}(s)$ to $F_{t+\delta t}^{+}(s)$.

(15) $$F_{t+\delta t}^{+}(s)=e^{-s\delta t}F_{t}^{+}(s)-F_{t}^{+}(s=\infty )+M(s)f_{t}.$$

In simple situations where it is sufficient to know pairwise associations between stimuli separated in time, this provides a complete model for constructing a timeline of the future.

## Credit assignment and Laplace temporal difference learning

Consider an experiment. In the control condition of this hypothetical experiment, the participant is presented with y followed by z at a delay of 5 s for N trials. In the experimental condition, during an initial phase of training, x is followed by z at a delay of 10s for some number of trials. After this initial traning, the participant is presented with x followed by y at a delay of 5s and then z 5 s after y for N trials. The number of pairings between y and z, and the delays between them, are identical in the two conditions so that ${M_{z}}^{y}(s)$ would be the same in the two conditions. However, whereas y is “solely responsible” for z in the control condition, x is also capable of predicting z in the experimental condition. We might expect the “credit” that y receives for predicting z to be less in the experimental condition and indeed, analogous effects are observed in temporal blocking experiments (e.g., Amundson & Miller, 2008).

**Figure 6.**
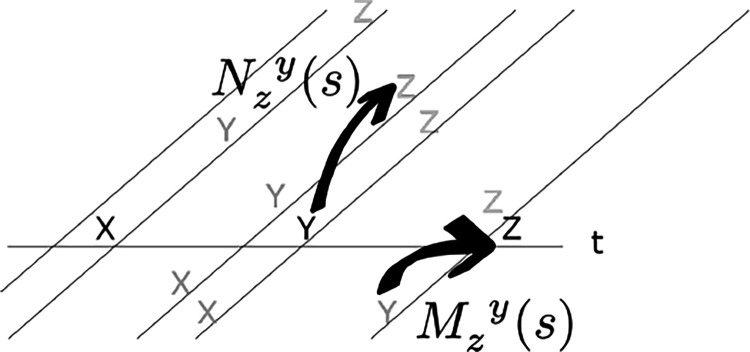
Whereas M(s) records the Laplace transform of the future that follows each item, N(s) records the expectation of the Laplace transform of the future predicted prior to that item. Consider a scenario with three consecutive items x, y, and z so that z is predicted prior to the presentation of y. ${M_{z}}^{y}(s)$ learns the connection between y in the past and z in the present. ${N_{z}}^{y}(s)$ learns the connection between y in the present and the prediction for z available just before y was presented. Comparing ${M_{z}}^{y}(s)$ to ${N_{z}}^{y}(s)$ allows one to estimate how much y is responsible for presentation of z.

Traditional RL makes sense of this phenomenon, and blocking more generally, by hypothesizing that plasticity at the time z is presented depends on how well it was predicted. However, in the present framework, in the experimental condition, z would be predicted an appropriate distance in the future at the moment x is presented. The two conditions thus differ in the degree to which z is predicted at the moment that y is presented. The basic strategy pursued here is to use conditions at the time of y to assess how much credit it should get for the occurrence of z, including the time of its presentation (Figure 6).

We assume that there is a prediction $F_{t}^{+}(s)$ available at all times, although this prediction can be zero. The “Laplace temporal difference” measure compares the true future that follows each stimulus, stored in M(s), to the future predicted just before each stimulus is presented. A second set of connections, N(s), records the average future available just before each item was observed. That is, for each stimulus y, $N^{y}(s)$ averages $F_{t}^{+}(s)$ observed at moments t such that $f_{t}=y$. The same strategy was used in Goh et al. (2022). We will see that this Laplace temporal difference is sensitive to the amount of information about both the identity and timing of future events. As such it provides a neurally-reasonable mechanism to estimate temporal contingency (Balsam & Gallistel, 2009; Gallistel, 2021b; Jeong et al., 2022).

### Estimating prediction independent of the present via N(s)

If y is presented at time t, then N(s) is updated as

(16) $$N^{y}(\rho ,s)\to \rho N^{y}(\rho ,s)+(1-\rho )F_{t}^{+}(s)$$

Following Eq. 13 we see that if ρ is a continuous variable, then $N^{y}(\rho ,s)$ is the Z-transform of the trial history of the predicted future available just prior to the moment y was presented. Note that, like M(ρ=1,s),N(ρ=1,s) can be decomposed into components corresponding to information about what symbols are predicted to occur and when. Definining $N_{\text{what}}=N(s=0)$, we can write $N(s)=N_{\text{what}}\cdot \overset{ˆ}{N}(s)$ much like we did for M(s).

To illustrate the properties of M(ρ,s) and N(ρ,s), let us restrict our attention to cases where at most three symbols x, y and z are presented in order on each trial. Let us refer to the time lags between symbols as random variables $\tau_{xy},\tau_{yz}$; on trials where all three symbols are observed $\tau_{xz}=\tau_{xy}+\tau_{yz}$ by assumption. We assume that the distributions are chosen such that the relative times of presentation do not overlap. We denote the probabilities of each symbol occuring on a trial such that P(z∣y) gives the conditional probability that z is observed on a trial given that y is also observed on that trial.

### Estimating three point correlation functions from M(ρ) and N(ρ)

A great deal of information can be extracted from the trial history encoded in M(ρ,s) and N(ρ,s). On a particular trial after y has been presented, we would like to compute the probability that z will be presented and at what time. M contains the two-point probability distribution of y and z. It would be preferable to predict the occurrence of z using the three-point probability distribution, taking into account the occurrence and timing of both x and y.

Because M(ρ,s) and N(ρ,s) contain information about the paired trial history, in principle we can extract information about the three-point correlation function. For instance, if z only occurs on trials on which both x and y are presented, then we should observe a positive correlation between the trial history encoded in ${M_{z}}^{y}(\rho ,s=0)$ and the trial history of predictions encoded in ${N_{z}}^{y}(\rho ,s=0)$. After all, if x is not presented on a particular trial, then it cannot predict z and the prediction written into ${N_{z}}^{y}(\rho ,s=0)$ on that trial will be zero. Similarly, one can imagine that the joint timing of the presentations of x and y predicts the timing of z. For instance, a positive correlation between $\tau_{xy}$ and $\tau_{yz}$ would be observed if x causes the future occurence of both y and z via some process whose rate varies across trials. If $\tau_{xy}$ is longer than average on a particular trial, then z will be predicted earlier than average on that trial. In this way, a positive correlation between $\tau_{xy}$ and $\tau_{yz}$ would manifest as a negative correlation across trials between the time at which z is observed and the time at which z is predicted. In principle this information can be extracted from ${{\overset{ˆ}{M}}_{z}}^{y}(\rho ,s)$ and ${{\overset{ˆ}{N}}_{z}}^{y}(\rho ,s)$.

### Associative contingency via Laplace temporal difference

A general model for predicting the future taking into account the past and the present, as well as the trial history encoded in M(ρ,s) and N(ρ,s) is beyond the scope of this paper. We will content ourselves, taking the limit as ρ→1, with a simple measure to estimate contingency between pairs of symbols. If y has no causal effect on the future that follows it, then $M^{y}(s)=N^{y}(s)$. This holds for what and when information taken separately. Let us define a simple measure of contingency between symbols, which we refer to as the Laplace temporal difference, as

(17) $$LTD(s)\equiv -log\frac{M(s)}{N(s)}=-log\frac{M_{\text{what}\;}}{N_{\text{what}\;}}-log\frac{\overset{ˆ}{M}(s)}{\overset{ˆ}{N}(s)}$$

where we understand the fractions of matrices on the right-hand-side to imply pointwise division. The ratio $\frac{M(s)}{N(s)}$ can be understood as an attempt to deconvolve the predictions that precede each symbol from the future that follows it. When a symbol does not predict the future occurrence of symbols that follow it, the deconvolution yields the Laplace transform of delta function at zero. The Laplace transform of δ(0)=1 for all s, so that LTD⁡(s) is zero for all s under these circumstances.

We will work out the properties of LTD⁡(s) in our simple case with three symbols to illustrate that it functions as a reasonable measure of contingency. We are interested in the contingency between y and z given the context provided by x.

From Eq. 12, we know that ${M_{z}}^{y}(s)=P(z|y)ℒ\left\{\tau_{yz}\right\}$. Because $\tau_{yz}=\tau_{xz}-\tau_{xy}$ by assumption, it is also true that ${M_{z}}^{y}(s)=P(z|y)ℒ\left\{\tau_{xz}-\tau_{xy}\right\}$. To compute ${N_{z}}^{y}(s)$ we first need to determine the prediction from x to z on each trial y is presented. Then we need to average that prediction over trials. Because ${N_{z}}^{y}(s)$ is only updated on trials where y is presented. x can only contribute to ${N_{z}}^{y}(s)$ when x is also presented, which occurs with probability P(x∣y). On each trial, right after x is presented the prediction for z is ${M_{z}}^{x}(s)=P(z|x)ℒ\left\{\tau_{xz}\right\}$. On each trial, at the time y is observed this contribution from x has evolved to $e^{s\left(t_{y}-t_{x}\right)}P(z|x)ℒ\left\{\tau_{xz}\right\}$, where $t_{y}-t_{x}$ is the value of the random variable $\tau_{xy}$ observed on that particular trial. Averaging over trials on which x is presented gives the Laplace transform of the convolution of $\tau_{xz}$ and $-\tau_{xy}$. Multiplying by the probability that x is presented at all we find

(18) $${N_{z}}^{y}(s)=P(z|x)P(x|y)ℒ\left\{\tau_{xz}*\left(-\tau_{xy}\right)\right\}(s).$$

Now,

(19) $$-log\frac{z^{\prime }M_{\text{what}\;}y}{z^{\prime }N_{\text{what}\;}y}=-log\frac{P(z|y)}{P(z|x)P(x|y)}$$

Equation 19 shows that the Laplace temporal difference identifies differences between future outcomes that are expected to follow the present stimulus—the numerator—and future outcomes that would be predicted by previous stimuli—the denominator. Although Eq. 19 is written for a single preceding symbol, this result obviously generalizes over mutually exclusive symbols that could precede $y,\sum_{x}\;P(z|x)P(x|y)$.

Considering temporal contingency, we find

(20) $$-log\frac{{{\hat{M}}_{z}}^{y}(s)}{{{\hat{N}}_{z}}^{y}(s)}=-\log\frac{ℒ\left\{\tau_{xz}-\tau_{xy}\right\}(s)}{ℒ\left\{\tau_{xz}*\left(-\tau_{xy}\right)\right\}(s)}$$

Recall that the expectation of the sum of two independent random variables is equal to their convolution. The ratio in Equation 20 compares the Laplace transform of the sum of two random variables to the Laplace transform of their convolution. If $\tau_{xz}$ and $-\tau_{xy}$ are independent, then the ratio is 1 and the right hand side is zero for all s. This demonstrates that LTD⁡(s) is sensitive to the temporal contingency between y and z.

## Neural predictions

Regions as widely separated as the cerebellum (Wagner & Luo, 2020; De Zeeuw, Lisberger, & Raymond, 2021), striatum (e.g., van der Meer & Redish, 2011), PFC (e.g., Rainer, Rao, & Miller, 1999; Ning, Bladon, & Hasselmo, 2022), OFC (e.g., Namboodiri et al., 2019; Schoenbaum, Chiba, & Gallagher, 1998; Young & Shapiro, 2011), hippocampus (Ferbinteanu & Shapiro, 2003; Duvelle, Grieves, & van der Meer, 2022) and thalamus (Komura et al., 2001) contain active representations that code for the future. One can find evidence of predictive signals extending over long periods of time that modulate firing in primary visual cortex (Gavornik & Bear, 2014; Kim, Homann, Tank, & Berry, 2019; Homann, Koay, Chen, Tank, & Berry, 2022; Yu et al., 2022). Prediction apparently involves a substantial proportion of the brain. Coordinating activity and plasticity over such a wide region would require careful synchronization (Hasselmo, Bodelón, & Wyble, 2002; Hamid, Frank, & Moore, 2021). The timescale of this synchronization, presumably on the order of 100 ms, fixes δt, places a bound on the fastest timescales 1/*s* that can be observed, and operationalizes the duration of the “present.”

Given the widespread nature of predictive signal, we will not attempt to map specific equations onto specific brain circuits. Rather we will illustrate the observable properties implied by these equations with an eye towards facilitating future empirical work. The predictions fall into two categories. One set of predictions describes properties of ongoing firing of neurons participating in Laplace and inverse spaces. Another set of predictions are a direct consequence of the properties of learned weights. We also briefly discuss the model in this paper in the context of recent empirical work on the computational basis of the dopamine signal (Jeong et al., 2022).

### Active firing neurons

This paper proposes the existence of neural manifolds to code for the identity and time of *future* events. The prediction is that there should be two related manifolds, one implementing the Laplace space and one implementing the inverse space. Previous neuroscientific work has shown evidence for Laplace and inverse spaces for a timeline for the past. The properties of the proposed neural manifolds for future time can be understood by analogy to the neural manifolds for the past.

#### Single-cell properties of neurons coding for the past.

So-called temporal context cells observed in the entorhinal cortex (Tsao et al., 2018; Bright et al., 2020) are triggered by a particular event and then relax exponentially back to baseline firing with a variety of time constants. The firing of temporal context cells is as one would expect for a population coding $F^{-}(s)$. So-called time cells observed in the hippocampus (Pastalkova et al., 2008; MacDonald et al., 2011; Taxidis et al., 2020; Shahbaba et al., 2022; Shikano, Ikegaya, & Sasaki, 2021; Schonhaut, Aghajan, Kahana, & Fried, 2022) and many other brain regions (e.g., Tiganj, Cromer, Roy, Miller, & Howard, 2018; Tiganj, Kim, Jung, & Howard, 2017; Mello, Soares, & Paton, 2015; Bakhurin et al., 2017; Akhlaghpour et al., 2016; Jin, Fujii, & Graybiel, 2009) fire sequentially as events recede into the past, as one would expect from neurons participating in $\tilde{f}(\overset{*}{\tau }<0)$. Time cells are consistent with qualitative and quantitative predictions of $\tilde{f}(\overset{*}{\tau }<0)$, including the conjecture that time constants are distributed along a logarithmic scale (Cao et al., 2022).

#### Single-cell and population-level properties of neurons coding for the past and the future.

**Figure 7.**
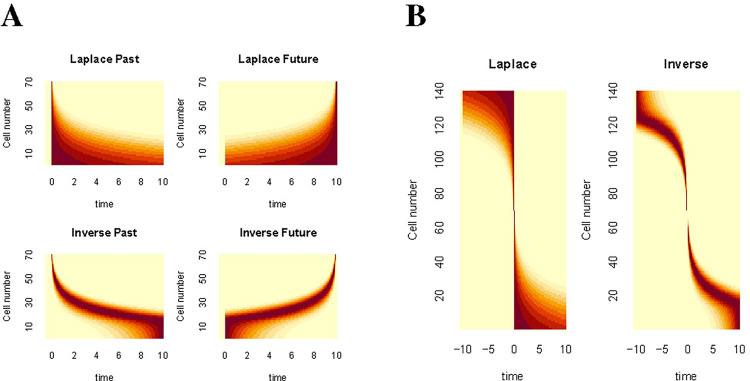
Predicted firing for Laplace and inverse spaces plotted as heatmaps. **A.** Consider an experiment in which x precedes y separated by 10 s. The top row shows firing as a function of time for cells in the Laplace space for the past (left) and the future (right). Note that the cells in $F_{t}^{-}(s)$ peak at time zero and then decay exponentially. In contrast cells in $F_{t}^{+}(s)$ peak at 10 s and ramp up exponentially. The bottom row shows firing as a function of time for cells in the Inverse space. **B.** Consider an experiment in which y is predicted to occur at time zero and then recedes into the past. Cells coding for both past and future are recorded together and sorted on the average time at which they fire. Left: For Laplace spaces, neurons in $F_{t}^{+}(s)$ are sorted to the top of the figure and neurons $F_{t}^{-}(s)$ are sorted to the bottom of the figure. Right: Inverse spaces show similar properties but give rise to a characteristic “pinwheel” shape.

In situations where the future can be predicted, $F^{+}(s)$ and $\tilde{f}(\overset{*}{\tau }>0)$ should behave as mirror images of the corresponding representations of the past. Figure 7A illustrates the firing of cells coding for a stimulus remembered in the past (left) and predicted in the future (right). Neurons participating in the Laplace space, sorted on their values of s, are shown in the top; neurons participating in the inverse space, sorted on their values of $\overset{*}{\tau }$ are shown on the bottom.

The firing of neurons constituting the Laplace space shows a characteristic shape when plotted as a function of time in this simple experiment. Neurons coding for the past are triggered shortly after presentation of the stimulus and then relax exponentially with a variety of rates. Neurons coding for the future ramp up, peaking as the predicted time of occurrence grows closer. The ramps have different characteristic time constants. Different populations are triggered by the presentation of different symbols (not shown) so that the identity of the remembered and predicted symbols as well as their timing can be decoded from populations coding $F^{-}(s)$ and $F^{+}(s)$. The largest value of 1/s in the figure is chosen to be a bit longer than the delay in the experiment, resulting in a subset of neurons that appear to fire more or less constantly throughout the delay (Enel et al., 2020).

The firing of neurons constituting the inverse space also shows a characteristic shape when plotted as a function of time in this simple experiment. Neurons tile the delay, with more cells firing early in the interval with more narrow receptive fields. The logarithmic compression of n results in a characteristic “backwards J” shape for the past and a mirror image “J” shape for the future. Again, different populations would code for different stimuli in the past and in the future (not shown) so that the identity of the remembered and predicted stimuli and their time from the present could be decoded from a population coding $f\left(\overset{*}{\tau }\right)$. Figure 7B shows firing that would be expected for a population that includes cells coding for the same stimulus, say y, both in the past and the future around the time of a predicted occurrence of that symbol.

#### Plausible anatomical locations for an internal future timeline.

This computational hypothesis should evaluated with carefully planned analyses. However, the published literature shows evidence that is at least roughly in line with the hypothesis of neural manifolds for future time. Firing that ramps systematically upward in anticipation of important outcomes including planned movements has been observed in (at least) mPFC (Henke et al., 2021), ALM (Inagaki, Inagaki, Romani, & Svoboda, 2018), and thalamus (Komura et al., 2001). Komura et al. (2001) showed evidence for ramping firing in the thalamus that codes for outcomes in a Pavlovian conditioning experiment. Preliminary evidence from secondary analyses suggest that there is a heterogeneity of time constants in ALM (Inagaki et al., 2018) and mPFC (Henke et al., 2021).

There is also circumstantial neurophysiological evidence for sequential firing leading to predicted future events as predicted by $\tilde{f}(\overset{*}{\tau }>0)$. Granule cells in cerebellum appear to fire in sequence in the time leading up to an anticipated reward (Wagner, Kim, Savall, Schnitzer, & Luo, 2017; Wagner & Luo, 2020). OFC may be another good candidate region to look for “future time cells.” OFC has long been argued by many authors to code for the identity of predicted outcomes (Hikosaka & Watanabe, 2000; Schoenbaum & Roesch, 2005; Mainen & Kepecs, 2009). More recently Enel et al. (2020) showed sequential activation in OFC during a task in which it was possible to predict the value of a reward that was delayed for several seconds. Finally, it should be noted that the properties of $\tilde{f}(\overset{*}{\tau }>0)$ are a temporal analog of spatial “distance-to-goal” cells observed in spatial navigation studies (Sarel, Finkelstein, Las, & Ulanovsky, 2017; Gauthier & Tank, 2018).

### Predictions from weight matrix M(ρ,s)

#### Properties of weights due to s.

**Figure 8.**
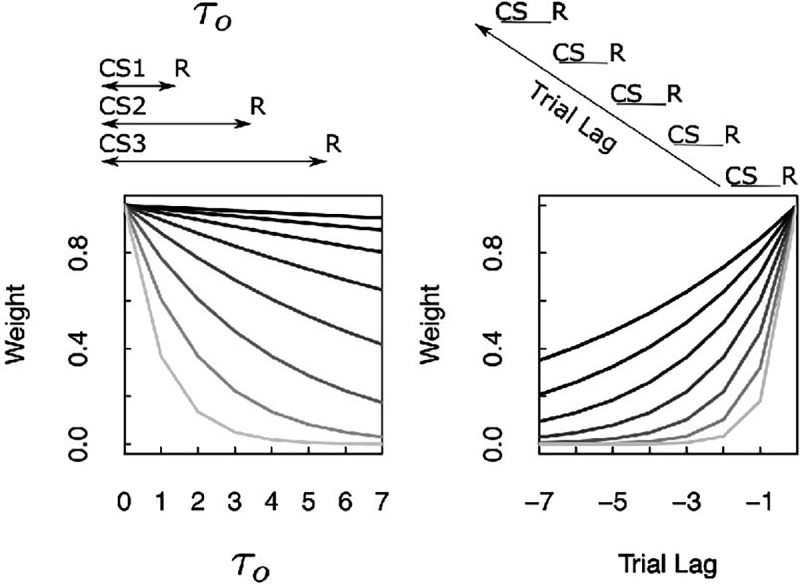
Neural predictions derived from properties of M(s). Left. Plot of the magnitude of the entry in $M_{r}(\rho =1,s)$ connecting each of the conditioned stimuli cs to the outcome R as a function of the $\tau_{o}$ corresponding to that cs. Different lines correspond to entries with different values of s. Weights corresponding to different values of s show exponential discounting as $\tau_{o}$ is manipulated, with a variety of discount rates. Right. Plot of the magnitude of M(ρ,s=0) associated with a single pairing of cs and R a certain number of trials in the past. Different lines show the results for different values of ρ. For clarity, these curves have been normalized such that they have the same value at trial lag zero.

Consider an experiment in which different symbols, denoted cs1, cs2, etc, precede an outcome R by a delay $\tau_{o}$. The value of $\tau_{o}$ changes across the different symbols (Figure 8A). Ignoring ρ for the moment, the strength of the connections from each cs to R depend on the value of $\tau_{o}$ for that stimulus and the value of s for each synapse: $e^{-s\tau_{o}}$. When a particular cs is presented at time t, the amount of information that flows along each synapse is $e^{-s\tau_{o}}$ and the pulse of input to $F_{t+\delta t}^{+}(s)-F_{t}^{+}(s)$ corresponding to the outcome is $e^{-s\tau_{o}}$.

Thus, considering each connection as a function of $\tau_{o}$, firing should go down exponentially as a function of $\tau_{o}$ with a rate constant that depends on the value of s. This pattern of results aligns well with results presented at SfN in 2022 (Masset, Malik, Kim, Bech Vilaseca, & Uchida, 2022). The experiment was constructed much as described above. Masset et al. (2022) measured the firing of dopamine neurons to different stimuli predicted reward delivery at different delays. It has long been known that firing of dopamine neurons, averaged over neurons, around the time of the conditioned stimulus goes down with delay (Fiorillo, Newsome, & Schultz, 2008). This study showed that there was a heterogeneity of exponential decay rates in the firing of dopamine neurons in this paradigm, much as illustrated in Fig. 8A. In the context of TDRL, this finding is also consistent with a continuous spectrum of exponential discount rates (Momennejad & Howard, 2018; Tano et al., 2020).

#### Properties of weights due to ρ.

A continuum of forgetting rates ρ predicts a range of trial history effects. Figure 8B shows the weights in M(ρ) over past trials that result from different values of ρ. This is simply $\rho^{i}$ where i is the trial recency with values normalized such that the weight at the most recent trial is 1. The weights M(ρ) record the trial history of reinforcement. The weights N(ρ) record the trial history of predicted outcomes. The difference between M(ρ) and N(ρ) as a function records the history of prediction violations. Many papers show dependence on previous trial outcomes in response to a cue stimulus in learning and decisionmaking experiments (Bernacchia, Seo, Lee, & Wang, 2011; Morcos & Harvey, 2016; Scott et al., 2017; Akrami, Kopec, Diamond, & Brody, 2018; Hattori, Danskin, Babic, Mlynaryk, & Komiyama, 2019; Hattori & Komiyama, 2022). These studies show history-dependent effects in a wide range of brain regions and often show a continuous spectrum of decay rates within a brain region (see especially Bernacchia et al., 2011). Notably, distributions of time constants for trial history effects cooccur with distributions of ongoing activity in multiple brain regions (Spitmaan, Seo, Lee, & Soltani, 2020).

### Dopamine and learning

The connection between TDRL and neuroscience related to dopamine has been one of the great triumphs of computational neuroscience (Schultz et al., 1997). The standard account is that the firing of dopamine neurons signals reward prediction error (RPE) which drives plasticity. Despite its remarkable success at predicting the findings of many behavioral and neurophysiological experiments, the RPE account has been under increasing strain over recent years. The standard account did not predict the existence of a number of striking effects, including increasing dopamine firing during delay under uncertainty (Fiorillo, Tobler, & Schultz, 2003), dopamine ramps in spatial experiments (Howe, Tierney, Sandberg, Phillips, & Graybiel, 2013), dopamine waves (Hamid et al., 2021), and heterogeneity of dopamine responses across neurons and brain regions (Dabney et al., 2020; Masset et al., 2022; W. Wei, Mohebi, & Berke, 2021). Recently Jeong et al. (2022) reported the results of several experiments that flatly contradict the standard model. These experiments were proposed to evaluate an alternative hypothesis for dopamine firing in the brain.

Jeong et al. (2022) propose that dopamine signals whether the current stimulus is a cause of reward. The model developed there, referred to as ANCCR, assesses the contingency between a stimulus and outcomes. LTD(*s*) measures the contingency—temporal and otherwise—between a symbol and possible outcomes. Both ANCCR and the framework developed in this paper are inspired by a similar critique of Rescorla-Wagner theory and TDRL (Gallistel, 2021b). In order to make a complete account of the experiments in the Jeong et al. (2022) paper, the current framework would have to be elaborated in several ways. In order to keep the calculations simple here we have assumed that each symbol can only occur once per trial. Consider the M(s) and N(s) that would result if this assumption were relaxed. If we define $p_{y}^{x}(\tau )$ as the probability that we will observe y in a small interval around t+τ given that we observed x at time t, we get

$${M_{z}}^{y}(s)=\int \;e^{-s\tau }{p_{z}}^{y}(\tau )d\tau =ℒ\left\{{p_{z}}^{y}\right\}(s).$$

Assuming we can pretend that it is acceptable to ignore the overlap between the distributions we would find

$$\begin{array}{r} {N_{z}}^{y}(s)=\sum_{x}\;\;\int \;e^{-s\tau }\left(p_{y}^{x}\#{p_{z}}^{x}\right)(\tau )d\tau \\ \;=ℒ\left\{{p_{y}}^{x}\#{p_{z}}^{x}\right\}(s). \end{array}$$

Where # signifies cross-correlation. This is closely analogous to Eqs. 19 and 20.

The expression for M(s) ends up giving expected number of observations of z that would follow y out to a timescale on the order of $1/s.{N_{z}}^{y}(s)$ gives the number of observations of z that were expected prior to observation of Y. It is thus possible to construct a measure of prospective contingency like that used in the Jeong et al. (2022) model. The current framework does not require one to specify an intrinsic timescale of association *a priori*.

## Discussion

This paper takes a phenomenological approach to computational neuroscience. The strategy is to write down equations that, if the brain could somehow obey them, would be consistent with a wide range of observed cognitive and neural phenomena. The phenomenological equations make concrete predictions that can be evaluated with cognitive and neurophysiological experiments. To the extent the predictions hold, the question of how the brain manages to obey these phenomenological equations could then become a separate subject of inquiry. The phenomenological equations require a number of capabilities of neural circuits, both at the level of synapses and in terms of ongoing neural activity. We make those explicit here.

### Circuit assumptions for synaptic weights

The connections M(ρ,s) and N(ρ,s) require that the brain uses continuous variables, s and ρ, to organize connections between many neurons, most likely spanning multiple brain regions. For the phenomenological equations to be viable, these continuous variables should be deeply embedded in the functional architecture of the brain. For instance, in order to invert the integral transforms, it is necessary to compute a derivative over these continuous variables. This suggests a gradient in these continuous variables should be anatomically identifiable. Conceivably anatomical gradients in gene expression and functional architecture (e.g., Phillips et al., 2019; Guo et al., 2021; Roy, Zhang, Halassa, & Feng, 2022) could generate anatomical gradients in s and/or ρ. Perhaps part of the function of traveling waves of activity such as theta oscillations (Lubenov & Siapas, 2009; Patel, Fujisawa, Berényi, Royer, & Buzsáki, 2012; Zhang & Jacobs, 2015) or dopamine waves (Hamid, Frank, & Moore, 2019) is to make anatomical gradients salient.

LTD⁡(s) is not intended as a literal description of a neural computation the brain could use to assess contingency between stimuli. However, the idea of assessing contingency by comparing the future that immediately precedes a stimulus to the future that follows it *should* be taken seriously. If brain oscillations are important in synchronizing the flow of information proposed in this framework, then perhaps information from M(s) and N(s) are available at different phases of an oscillation. Perhaps this consistent relationship temporal combined with spike timing dependent plasticity (Bi & Poo, 1998; Dan & Poo, 2004) somehow facilitates computation of the contingency between the present and the future.

### Circuit assumptions for ongoing activity

At the neural level, this framework assumes the existence of circuits that can maintain activity of a Laplace neural manifold over time. There is evidence that the brain has found some solution to this problem, at least for past time and at least in the entorhinal cortex (Tsao et al., 2018; Bright et al., 2020). Exponential growth of firing, as proposed by Eq. 15 seems on its face to be a computationally risky proposition. However, this proposal does create testable predictions. Moreover, firing rates that increase monotonically in time are widely observed. For instance border cells as an animal approaches a barrier (Solstad, Boccara, Kropff, Moser, & Moser, 2008) and evidence accumulation cells (Roitman & Shadlen, 2002) both increase monotonically. If this monotonic increase in firing reflects movement of an edge along a Laplace neural manifold, the characteristic time scale of the increase should be heterogeneous across neurons. If the brain has access to a circuit with paired α’s, it could reuse this circuit to construct cognitive models for spatial navigation (Howard et al., 2014), evidence accumulation (Howard et al., 2018), and perhaps cognitive computation more broadly (Howard & Hasselmo, 2020). Consistent with this hypothesis, monotonic cells in spatial navigation and evidence accumulation—border cells and evidence accumulation cells—have sequential analogues (Wilson & McNaughton, 1993; Morcos & Harvey, 2016; Koay, Charles, Thiberge, Brody, & Tank, 2022) as one would expect if they reflect a Laplace space that is coupled with an inverse space.

Perhaps part of the solution to implementing these equations in the brain is to restrict the kinds of functions that can be represented over the Laplace manifold. Perhaps a continuous attractor network that can maintain and evolve the Laplace transform of a single delta function per basis vector would be straightforward to construct. In this case, each component of $F_{t}^{-}(s)$ and $F_{t}^{+}(s)$ would be at any moment the Laplace transform of a delta function; M(s) and N(s) would still be able to store distributions over multiple presentations. In this case perhaps $F_{t+\delta t}^{+}(s)$ could update by sampling from the distribution expressed by $M(s)f_{t}$. Perhaps predictions are updated in the more general case by sampling from a super-position of the previous prediction $F_{t}^{+}(s)$ and the future that follows the current item $Msf_{t}$ masked by $N(s)f_{t}$.

### What about control?

RL algorithms have been successful in AI application because of their ability to learn policies to control actions in the absence of explicit supervision (Watkins & Dayan, 1992; Sutton & Barto, 2018). The current framework does not include a deep connection to control theory. It is conceivable that the current framework could be integrated into existing deep network approaches to RL. Perhaps it is possible to learn an embedding that maps continuous features onto symbols then control actions using existing methods from deep RL. Another possibility is to develop a control theory in the Laplace domain. Indeed, this is how control theory problems are typically solved analytically (Ogata, 1970). Indeed, there is some evidence that control systems in neurobiology, for instance gaze stabilization in the oculumotor system, make use of multiple time constants over several orders of magnitude (Miri, Bhasin, Aksay, Tank, & Goldman, 2022). Perhaps a continuous set of time constants, as required for the Laplace neural manifolds used here, may enable brains to make use of diversity enabled sweet spots (Nakahira, Liu, Sejnowski, & Doyle, 2021).

It should be possible to extend the current framework to multiple dimensions beyond time, including real space and abstract spaces (Howard et al., 2014, 2018). Properties of the Laplace domain enable data-independent operators that enable efficient computation (Howard & Hasselmo, 2020). For instance, given that a state of a Laplace neural manifold is Laplace transform of a function, we can construct the Laplace transform of the translated function (Eq. 7, see also (Shankar, Singh, & Howard, 2016)). Critically, the translation operator is independent of the function to be translated. Restricting our attention to Laplace transforms of delta functions, we can construct the sum or difference using convolution and cross correlation respectively (Howard et al., 2015; Howard & Hasselmo, 2020). The binary operators for addition and subtraction also do not need to be learned. Perhaps the control theory that governs behavior is analogous to generic spatial navigation in a continuous space.

### Scale-covariance as a design goal

Because the s values are sampled along a logarithmic scale, all of the quantities in this paper are scale-covariant. Rescaling time, taking $\tau_{xz}\to a\tau_{xz},\tau_{xy}\to a\tau_{xy}$, etc, simply takes s→s/a. Because the s values are chosen in a geometric series, rescaling time simply translates along the n axis. All the components of the model, $F^{-},F^{+},M,N$, and LTD⁡(s), all use the same kind of logarithmic scale for time. This means that rescaling time simply translates all the components of the entire model, up to a scaling factor. All of the components of the model are thus time scale-covariant, responding to rescaling time with a translation over cell number. Thus any measure that integrates over n (and is not subject to edge effects) is scale-invariant.

Empirically, there is not a characteristic time scale to associative learning (Balsam & Gallistel, 2009; Gershman, 2022); any model that requires choice of a time scale for learning to proceed is thus incorrect. Logarithmic time scales are observed neurally (Cao et al., 2022; Guo et al., 2021). Logarithmic time scales can be understood as a commitment to a world with power law statistics (X.-X. Wei & Stocker, 2012; Piantadosi, 2016) or as an attempt to function in many different environments without a strong prior on the time scales it will encounter (Howard & Shankar, 2018).

Recent work has shown that the use of logarithmic time scales enables scale-invariant CNNs for vision (Jansson & Lindeberg, 2021) and audition (Jacques, Tiganj, Sarkar, Howard, & Sederberg, 2022). For instance, Jacques et al. (2022) trained deep CNNs to categorize spoken digits. When tested on digits presented at very different speeds than the training examples (imagine someone saying the word “seven” stretched over four seconds), the deep CNN with a logarithmic time axis generalized perfectly. Rescaling time translates the neural representation at each layer; convolution is translation equivariant; including a maxpool operation over the convolutional layer renders the entire CNN translation-invariant. Time is not only important in speech perception (e.g., Lerner, Honey, Katkov, & Hasson, 2014) but vision as well (Russ, Koyano, Day-Cooney, Perwez, & Leopold, 2022) suggesting that these ideas can be incorporated into a wide range of sensory systems.

## Footnotes

1 Because of the sign conventions adopted here, $F_{t}^{-}(s)$ is the Laplace transform of $f_{t}(-\tau )$ whereas $F_{t}^{+}(s)$ is the transform of $f_{t}(\tau )$. Viewed in this light it is more precise to think of Eq. 10 as learning the Laplace transform of the cross-correlation between the present and the past.
